# Parenclitic Network Mapping Identifies Response to Targeted Albumin Therapy in Patients Hospitalized With Decompensated Cirrhosis

**DOI:** 10.14309/ctg.0000000000000587

**Published:** 2023-04-05

**Authors:** Tope Oyelade, Ewan Forrest, Kevin P. Moore, Alastair O'Brien, Ali R. Mani

**Affiliations:** 1 Division of Medicine, Institute for Liver and Digestive Health, UCL, London, UK;; 2 Division of Medicine, Network Physiology Laboratory, UCL, London, UK;; 3 Department of Gastroenterology, Glasgow Royal Infirmary, Glasgow, UK.

## Abstract

The efficacy of targeted albumin therapy in the management of decompensatory events in cirrhosis is unclear, with different reports showing conflicting results. It is possible that only certain subgroups of patients may benefit from targeted albumin administration. However, extensive conventional subgroup analyses have not yet identified these subgroups. Albumin is an important regulator of physiological networks and may interact with homeostatic mechanism differently in patients according to the integrity of their physiological network. In this study, we aimed to assess the value of network mapping in predicting response to targeted albumin therapy in patients with cirrhosis.

**METHODS:**

This is a substudy of the ATTIRE trial, a multicenter randomized trial conducted to assess the effect of targeted albumin therapy in cirrhosis. Baseline serum bilirubin, albumin, sodium, creatinine, CRP, white cell count (WCC), international normalized ratio, heart rate, and blood pressure of 777 patients followed up for 6 months were used for network mapping using parenclitic analysis. Parenclitic network analysis involves measuring the deviation of each patient from the existing network of physiological interactions in a reference population.

**RESULTS:**

Overall network connectivity and deviations along the WCC-CRP axis predicted 6-month survival independent of age and model for end-stage liver disease in the standard care arm. Patients with lower deviation along the WCC-CRP axis showed lower survival in response to targeted albumin administration over a 6-month follow-up period. Likewise, patients with higher overall physiological connectivity survived significantly less than the standard care group after targeted albumin infusion.

**DISCUSSION:**

The parenclitic network mapping can predict the survival of patients with cirrhosis and identify patient subgroups that do not benefit from targeted albumin therapy.

## Graphical abstract

**Figure:**
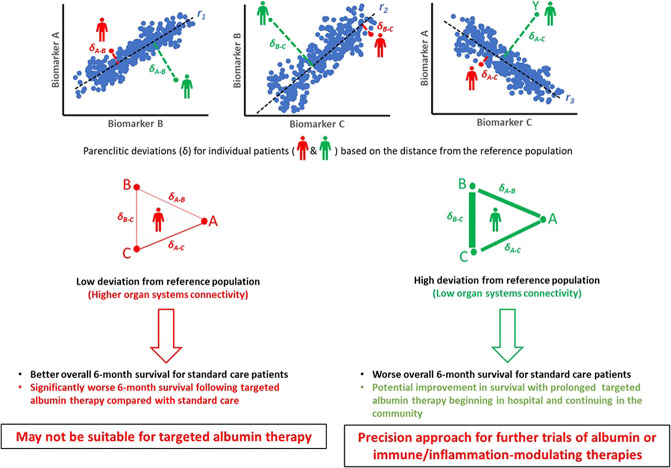

## INTRODUCTION

There has been an epidemic of liver disease during the past 50 years in the United Kingdom, with a 4-fold increase in liver-related deaths (1). This is now the leading cause of death in people aged 35–49 years and the second leading cause of working years lost in Europe (2). Many patients present late in the disease course with advanced cirrhosis, which confers a grim mortality (3,4). At this stage, the only life-prolonging treatment is liver transplantation, which is a limited and costly intervention. Albumin infusions have long been used for the management of complications of cirrhosis to improve plasma oncotic pressure and alleviate ascites and peripheral edema (5,6). Current international guidelines recommend use after large-volume paracentesis in patients with spontaneous bacterial peritonitis and hepatorenal syndrome (7,8), and several studies have demonstrated potential beneficial immune-mediated/anti-inflammatory properties (9–12). Furthermore, Bajaj et al reported that the low serum albumin level was significantly linked with an increased risk of death among hospitalized patients with cirrhosis and infection (13). However, albumin use outside of recommended indications remains a contentious clinical concept (14–19). Indeed, the ATTIRE clinical trial of targeted albumin therapy did not show benefit over standard care, and patients in the albumin group, who received 10 times (median of 200 g during hospitalization) as much albumin as those in standard care (20 g), had more severe or life-threatening serious adverse events, especially pulmonary edema or fluid overload (20,21).

Other clinical trials of albumin infusion have shown conflicting results. For instance, a meta-analysis of albumin use in patients with spontaneous bacterial peritonitis reported significant benefit (22) that was not seen in patients with other infections (23), and this latter trial was terminated because of lethal pulmonary edema associated with albumin (23). Taken together, these data suggest that certain subgroups of patients with advanced cirrhosis may benefit from targeted albumin therapy, but in others, this may cause harm. Yet, extensive conventional subgroup analyses of the ATTIRE data set did not identify patients who benefited, and there are no current biomarkers to guide albumin therapy. We therefore undertook an unsupervised analytic approach.

Healthy individuals show a high degree of functional connectivity between physiological organ systems. Disruption of organ system coupling is a hallmark of complex diseases, and recent studies showed that reduced network connectivity was linked with poor survival in patients with sepsis (24) and poor survival regardless of the severity of liver disease in patients with cirrhosis (25). This approach is novel because most prognostic indicators consider individual organ systems as separate units and do not reference their complex and nonlinear interactions.

Targeted albumin infusion has the potential to challenge the physiological network through alteration of oncotic pressure, plasma volume, glomerular filtration rate (GFR), and transportation of various physiological molecules. Therefore, the assessment of physiological interaction as a network may allow for a more precise prediction of patients' response or outcome. The parenclitic network mapping allows network analysis of static, baseline clinical variables on the individual patient level (26). Our group recently applied this method to a cohort of patients with cirrhosis using routine baseline clinical data and found that physiological network mapping can predict survival independent of the severity of liver disease as measured by the model for end-stage liver disease (MELD) (27). We hypothesized that parenclitic network analysis of routine clinical variables may provide valuable insight into the organ system disconnections associated with albumin treatment response and mortality in patients hospitalized with decompensated cirrhosis. We therefore used network analysis to assess organ system connectivity of individual patients based on their routinely available laboratory and clinical variables at trial entry, comparing treatment groups to identify baseline characteristics that precited a good or poor survival outcome to targeted albumin therapy.

## METHODS

### Study population

This is a substudy (and extension) of the ATTIRE trial, which was a randomized controlled trial of targeted albumin infusions vs standard care involving 777 patients hospitalized with decompensated cirrhosis from 35 hospitals across England, Wales, and Scotland (2016–2019) (20). There were no differences in baseline characteristics and outcome between treatment groups. Parenclitic network analysis was performed using routine clinical variables of patients in the standard care group alone to exclude any influences of targeted albumin treatment. Clinical variables analyzed include serum albumin (Alb), total bilirubin (Bil), international normalized ratio (INR), serum creatinine (Cr), serum sodium (Na), white cell count (WCC), C-reactive protein (CRP), mean arterial pressure, and heart rate (HR). These values were collected at trial entry, which was on average day 2 of hospitalization. Also, the variables were used for network analysis because they represent various physiological pathways directly and indirectly linked with liver function and are standard variables often measured in patients with liver cirrhosis. Furthermore, a previous report using random forest machine learning algorithms indicated that the chosen variables have weight in mortality prediction (25). Patients were grouped as survivors and nonsurvivors based on survival status after 6 months of the follow-up period. The original trial (ATTIRE) was conducted in accordance with both the Declarations of Helsinki and was approved by the London-Brent Research Ethics Committee and the Medicines and Healthcare Products Regulatory Agency (20).

### Parenclitic network analysis

**Figure 1.**
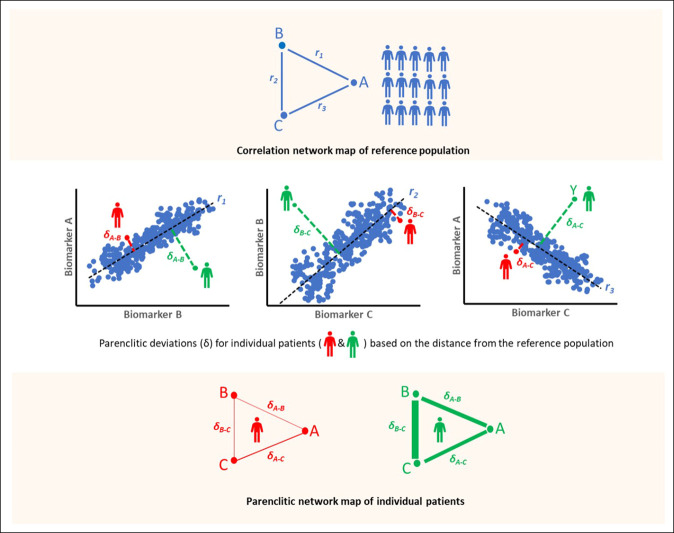
Schematic representation of the network mapping method used in this study for the reference population (top panel) and individual patients (lower panel). Top panel: The correlation between a pair of biomarkers (e.g., A-B, A-C, or B-C) was used for network mapping of the reference population (i.e., survivors in the standard care group). The blue dots represent individual reference data, and the black regression lines represent the expected relationship models (r_1_, r_2_, and r_3_ represent statistically significant correlation coefficients). Middle panel: To map the network of individual patients, a parenclitic approach was used. Parenclitic analysis measures the deviations of an individual patient from the expected relationship between variables in the reference population. In other words, parenclitic deviation indicates how far an individual biomarker level is from the expected model. In this example, the patient represented in red is closer to the reference population than the patient represented in green, in terms of the correlation between the biomarkers. Hence, the green patient has higher parenclitic deviation (δ) than the red patient. Lower panel: The resulting parenclitic network map of nodes A, B, and C is presented with edges weighted (in terms of thickness) according to the magnitude of deviations from the models for 2 individual patients (red and green). Higher thickness in edges in the green patient shows higher parenclitic deviation and thus less functional connectivity between biomarkers. The red patient has less parenclitic deviation, which means a closer association with the reference model and higher functional connectivity between biomarkers.

Parenclitic network analysis is a novel static network analytical method (26) that allows network mapping of individual datapoint from models built from a reference population with expected conditions (healthy and survivors). The deviation of individuals' characteristics from expectation is used to weigh the connection between variables for that individual (Figure 1). For a comprehensive description of the parenclitic network analysis in cirrhosis, please see the study by Zhang et al (27).

The ATTIRE survivors' population at 6 months was used as the reference population for the development of the parenclitic model, and deviations from this model for individual patients (survivors and nonsurvivors) were computed and used to weigh the correlation network map of clinical variables. Furthermore, the parenclitic indexes, including deviations along variable pairs and a global network topology of all patients (treatment and standard care), were computed using an in-house code in MATLAB (MathWorks, CA). For the measurement of global network topology, indexes such as network diameter, mean centrality, and shortest path length were calculated. Please see Supplementary Table S1 (Supplementary Digital Content, http://links.lww.com/CTG/A931) for the definition of network indexes used in this study. In general, higher global parenclitic network topology indexes such as diameter, mean centrality, and shortest path length indicate lower connectivity among all components of the network (Figure 1).

To validate the prognostic value of the parenclitic indexes, a split technique was used whereby ∼50% of patients in the standard treatment arm were randomly selected (training sample, n = 194), and the remainder were used as the validation sample (n = 203). Survivors in the training group were used as a model for the calculation of the coefficients that were used for the calculation of parenclitic deviations in the validation group. Statistical analysis was performed to test whether the prognostic values persist following the random split in the validation sample. This is to confirm whether the result generated was independent of the population studied.

### Statistical analysis

Statistical analysis was performed using Stata statistical software (Stata/MP, Version 17.0) and SPSS Statistics 26 (IBM Corp., Armonk, NY), with data presented as median and interquartile range (IQR) or mean ± SD. A 2-tailed *P* value of <0.05 was defined as statistical significance in all analyses. The Bonferroni correction was applied when multiple hypotheses were tested within a single analysis.

Initially, we performed the Mann-Whitney *U* test to compare the median of computed parenclitic variables, including the deviations along physiological axes (denoted as δ in this report) and global network topology indexes, for the survivor and nonsurvivor groups. Significantly different parenclitic variables were then tested for prognostic value by computing univariate and then multivariate Cox regression controlling for MELD and age. Parenclitic variables with independent predictive values were combined with MELD to produce a combinatory prognostic index based on coefficients of bivariate Cox regressions as follows: composite index = β_1_ × MELD + β_2_ × δ, where β_1_ and β_2_ are, respectively, the regression coefficient of MELD and parenclitic indexes in the bivariate Cox model.

Receiver operating curve (ROC) analyses were performed and the area under the curve (AUC) computed for individual and combinatory indexes to generate cutoffs. The specificity and sensitivity of resulting cutoffs were then used to generate the positive and negative predictive values based on Bayesian priors (% mortality) (28).

To test whether parenclitic indexes may differentiate between survivors and nonsurvivors at 6 months after targeted albumin treatment, we performed a multivariate Cox regression including patients' treatment arm (albumin or standard care) and each of the parenclitic indexes as interacting variables. The cutoff of parenclitic indexes that showed significant interaction with treatment in predicting survival was then used to categorize each patient into “1” if ≥cutoff and “0” if otherwise. The treatment arm of patients was then used to plot ROC curves to assess the 6-month survival grouped by the cutoffs of the significant parenclitic indexes.

To assess possible improvement in prognostic performance of MELD due to the addition of parenclitic indexes, Brier scores, integrated discrimination improvement (IDI), and net reclassification indexes (NRIs) were computed on Stata statistical software. The Brier score provides the mean of the squared distance between observed and predicted risks of event (mortality) for the individual patient. Generally, the lower the Brier score, the better the predictive model (29). IDI and NRI measure the improvement of a binary predictive model because of the addition of new variables (30).

## RESULTS

### Patient characteristics at ATTIRE trial entry

A total of 397 of 777 (51%) patients received standard care and were subjected to parenclitic analysis. During the 6-month follow-up period, 119 (30%) of these patients died. Table 1 presents the demographics and clinical characteristics of the study population.

**Table 1.** Demographic and baseline clinical variables in the study population

Characteristics	Survivors (n = 278)	Nonsurvivors (n = 119)	*P* value
Mean age, yr (SD)	52.3 (10.5)	57.1 (10.0)	**<0.001**
Male sex, n (%)	205 (74)	89 (75)	0.973
Cause of cirrhosis, n (%)			
Alcohol	247 (88.9)	104 (87.4)	
Hepatitis C	26 (9.4)	9 (7.6)	
Nonalcoholic fatty liver disease	19 (6.8)	10 (8.4)	
MELD score (median, IQR)	17.96 (14.6–22.4)	22.04	**<0.001**
Physiological variables			
Creatinine level, μmol/L	67 (56–85)	81 (58–123.5)	**0.001**
Bilirubin level, μmol/L	90 (40.5–144.5)	109.5 (59.5–222.5)	**0.002**
Serum albumin, g/L	24 (21–26)	24 (21–25.8)	0.768
International normalized ratio	1.6 (1.4–1.9)	1.8 (1.5–2.1)	**<0.001**
White cell count, 10^9^/L	6.8 (4.9–9.7)	8.8 (6.4–13.8)	**<0.001**
Serum sodium, mmol/L	134 (130–137)	131 (127–135)	**<0.001**
C-reactive protein, mg/L	21 (10–42)	36.5 (16–66)	**<0.001**
Heart rate, beats/min	89 (79–100)	92 (81–105)	**0.044**
Mean arterial pressure, mm Hg	83.3 (75.5–91.5)	81.7 (74.3–90.4)	0.241

The bold entries indicate *P* values that are statistically significant.

HCV, hepatitis C virus; IQR, interquartile range; MELD, model for end-stage liver disease; NAFLD, nonalcoholic fatty liver disease.

### Parenclitic indexes at ATTIRE trial baseline (prealbumin treatment) predict survivors and nonsurvivors at 6 months

**Figure 2.**
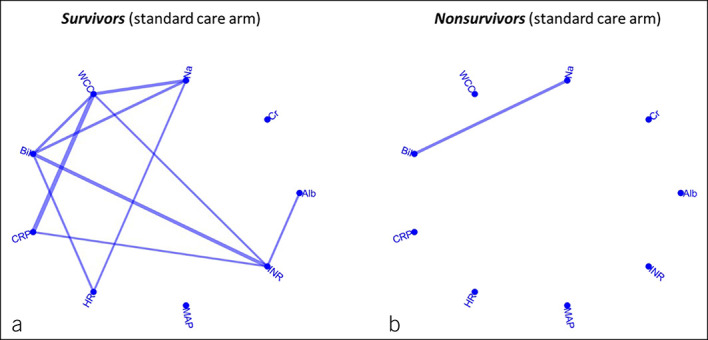
Correlation network map of survivors (**a**) and nonsurvivors (**b**) after a 6-month follow-up period of patients with decompensated cirrhosis under standard treatment. Each link shows a statistically significant correlation between 2 biomarkers after Bonferroni correlation for the total number of multiple comparisons. Alb, serum albumin; Bil, total bilirubin; CRP, C-reactive protein; HR, heart rate; INR, international normalized ratio; Na, serum sodium; WCC, white cell count.

The correlation network maps show that survivors had a significantly higher association between the baseline clinical variables compared with nonsurvivors (Figure 2). Overall, patients who survived for at least 6 months showed a significantly lower parenclitic deviation between baseline clinical variables compared with nonsurvivors in all indexes of the network topology (centrality, shortest path length, efficiency, and diameter). Specifically, there was a significantly lower parenclitic deviation along the WCC-CRP axis in survivors (Table 2).

**Table 2.** Differences in parenclitic indexes between survivors and nonsurvivors who received standard care in the studied population

Variables	Survivors	Nonsurvivors	*P* value
δ (WCC-Na)	1.492 (0.784–2.548)	0.254 (0.110–0.472)	0.063
δ (Bil-Na)	3.381 (1.797–6.244)	1.968 (0.809–3.162)	0.604
δ (INR-Alb)	0.276 (0.139–0.420)	3.563 (2.086–6.494)	0.981
δ (HR-Na)	2.736 (1.157–4.832)	2.710 (1.594–5.236)	0.315
δ (Bil-WCC)	2.500 (1.279–4.219)	3.155 (1.235–5.268)	0.089
δ (WCC-CRP)	**2.050 (0.980–3.810)**	**3.070 (1.330–5.320)**	**0.001**
δ (INR-WCC)	0.253 (0.120–0.430)	0.305 (0.144–0.506)	0.082
δ (HR-Bil)	9.102 (4.887–15.052)	10.451 (4.847–17.969)	0.138
δ (INR_Bil)	0.251 (0.125–0.433)	0.299 (0.129–0.498)	0.097
Mean centrality	**6.440 (4.660–8.320)**	**7.670 (4.890–10.610)**	**0.001**
Mean shortest path	**3.450 (2.520–4.670)**	**4.160 (2.800–6.000)**	**<0.001**
Diameter	**12.320 (8.550–18.990)**	**15.280 (9.760–22.270)**	**0.005**
Age	**51.858 (45.398–58.888)**	**56.320 (50.360–64.556)**	**<0.001**
MELD	**17.955 (14.467–22.385)**	**21.917 (17.690–26.328)**	**<0.001**

The bold entries indicate parenclitic indexes that significantly predict survival.

δ, deviation along an axis; Alb, serum albumin; Bil, total bilirubin; CRP, C-reactive protein; HR, heart rate; INR, international normalized ratio; MELD, model for end-stage liver disease; Na, serum sodium; WCC, white cell count.

### Prognostic values of baseline parenclitic indexes to predict 6-month outcome

According to our univariate Cox regression analysis, greater parenclitic deviations along WCC-Na, Bil-WCC, WCC-CRP, HR-Bil, and INR-Bil axes were all associated with an increased risk of 6-month mortality (Table 3). Likewise, higher measures of parenclitic network topology (i.e., mean centrality, mean shortest path length, and diameter) were associated with an increased risk of mortality up to 6 months (Table 3). Expectedly, a 1-unit increase in MELD resulted in 7.7% increase in the risk of 6-month mortality (hazard ratio: 1.077 (1.051–1.104), *P* < 0.001).

**Table 3.** Result of univariate Cox regression analysis for parenclitic indexes

Variables	β	SE	*P* value	Hazard ratio (95% CI)
δ (INR-Alb)	0.376	0.235	0.11	1.456 (0.919–2.307)
δ (WCC-Na)	**0.128**	**0.046**	**0.005**	**1.137 (1.039–1.243)**
δ (Bil-Na)	0.022	0.023	0.351	1.022 (0.976–1.07)
δ (HR_Na)	0.052	0.029	0.071	1.054 (0.995–1.115)
δ (Bil-WCC)	**0.075**	**0.021**	**<0.001**	**1.077 (1.033–1.124)**
δ (WCC-CRP)	**0.12**	**0.026**	**<0.001**	**1.128 (1.072–1.186)**
**δ (INR-WCC)**	**0.476**	**0.214**	**0.026**	**1.609 (1.058–2.448)**
δ (HR-Bil)	**0.024**	**0.009**	**0.008**	**1.024 (1.006–1.043)**
δ (INR-Bil)	**0.41**	**0.192**	**0.033**	**1.507 (1.034–2.197)**
Mean centrality	**0.137**	**0.029**	**<0.001**	**1.146 (1.083–1.214)**
Mean shortest path	**0.213**	**0.044**	**<0.001**	**1.237 (1.134–1.349)**
Diameter	**0.032**	**0.008**	**<0.001**	**1.033 (1.016–1.05)**
MELD	**0.074**	**0.013**	**<0.001**	**1.077 (1.051–1.104)**

The bold entries indicate parenclitic indexes that significantly predict survival.

δ, deviation along an axis; Alb, serum albumin; Bil, total bilirubin; CRP, C-reactive protein; HR, heart rate; INR, international normalized ratio; MELD, model for end-stage liver disease; Na, serum sodium; WCC, white cell count.

### Independent prognostic values of baseline parenclitic indexes to predict 6-month outcome

Multivariate Cox regression was performed to assess whether parenclitic indexes that individually predicted 6-month mortality had a prognostic value independent of age and MELD. Parenclitic deviations along the WCC-CRP axis (hazard ratio, 95% CI = 1.112, 1.053–1.174) and Bil-WCC axis (hazard ratio, 95% CI = 1.062, 1.017–1.108) significantly predicted outcome independent of age and MELD of patients at trial entry (Table 4). Mean centrality (hazard ratio, 95% CI = 1.094, 1.030–1.162), mean shortest path length (hazard ratio, 95% CI = 1.163, 1.059–1.276), and network diameter (hazard ratio, 95% CI = 1.025, 1.007–1.043) also predicted survival independent of age and MELD.

**Table 4.** Prognostic values of parenclitic indexes independent of age and MELD at admission

Variables	β	SE	*P* value	HR (95% CI)
**δ (WCC-CRP)**	**0.106**	**0.028**	**<0.001**	**1.112 (1.053–1.174)**
MELD	0.091	0.014	<0.001	1.095 (1.066–1.125)
Age	0.050	0.009	<0.001	1.051 (1.032–1.070)
δ (WCC-Na)	0.067	0.047	0.151	1.07 (0.976–1.173)
MELD	0.083	0.013	<0.001	1.086 (1.059–1.114)
Age	0.046	0.009	<0.001	1.047 (1.029–1.066)
δ (Bil-WCC)	**0.06**	**0.022**	**0.006**	**1.062 (1.017–1.108)**
MELD	0.083	0.013	<0.001	1.087 (1.06–1.114)
Age	0.047	0.009	<0.001	1.048 (1.03–1.066)
δ (HR-Bil)	0.014	0.009	0.131	1.014 (0.996–1.033)
MELD	0.084	0.013	<0.001	1.087 (1.061–1.115)
Age	0.046	0.009	<0.001	1.047 (1.029–1.065)
δ (INR-Bil)	−0.327	0.219	0.135	0.721 (0.469–1.107)
MELD	0.1	0.015	<0.001	1.106 (1.073–1.139)
Age	0.05	0.009	<0.001	1.051 (1.033–1.069)
Mean centrality	**0.090**	**0.031**	**0.004**	**1.094 (1.030–1.162)**
MELD	0.084	0.014	<0.001	1.088 (1.059–1.119)
Age	0.046	0.009	<0.001	1.047 (1.028–1.066)
Mean shortest path	**0.151**	**0.048**	**0.002**	**1.163 (1.059–1.276)**
MELD	0.085	0.014	<0.001	1.089 (1.059–1.119)
Age	0.046	0.009	<0.001	1.047 (1.028–1.066)
Diameter	**0.024**	**0.009**	**0.006**	**1.025 (1.007–1.043)**
MELD	0.091	0.014	<0.001	1.096 (1.066–1.126)
Age	0.047	0.009	<0.001	1.048 (1.029–1.068)

The bold entries indicate parenclitic indexes that significantly predict survival.

δ, deviation along an axis; Alb, serum albumin; Bil, total bilirubin; CRP, C-reactive protein; HR, heart rate; INR, international normalized ratio; MELD, model for end-stage liver disease; Na, serum sodium; WCC, white cell count.

The results of a parenclitic network analysis of a split sample (randomly selected patients) extracted from the study population showed similar correlation network maps of biomarkers (see Supplementary Figures S1 and S2, Supplementary Digital Content, http://links.lww.com/CTG/A931), and parenclitic deviations of the validation sample (203 random patients) calculated from the training sample (194 random patients) demonstrated similar results to the original findings using the overall sample (see Supplementary Tables S2 and S3, Supplementary Digital Content, http://links.lww.com/CTG/A931). Specifically, parenclitic deviation along the WCC-CRP axis as well as the mean shortest path length and diameter predicted 6-month survival independent of MELD and age of patients in this validation subset (see Supplementary Tables S2 and S3, Supplementary Digital Content, http://links.lww.com/CTG/A931).

### Receiver operating characteristic curve values of baseline parenclitic indexes to predict 6-month outcome

The area under the receiver operating characteristic (ROC) curves (AUC), cutoffs, and the sensitivity, specificity, positive and negative predictive values, and Brier scores of these cutoffs are presented in Table 5. Combining parenclitic indexes with MELD (composite indexes) consistently improved the AUC by up to 7% (0.709 vs 0.664) and approximately increased the positive predictive value of the optimum cutoff by 20% (40.59% vs 48.23%). Furthermore, Brier scores show that adding indexes of the parenclitic network improves the predictive performance of MELD (Table 5). Result from IDI and NRI analysis also shows an increased prognostic value by adding parenclitic network indexes to MELD (Table 6).

**Table 5.** Area on the ROC curves, sensitivity, specificity, PPV, NPV, and Brier score of parenclitic indexes in combination with MELD compared with MELD alone

Variables	AUC	*P* value	Cutoff	Sensitivity	Specificity	% increase in AUC vs MELD	PPV	NPV	Brier score
MELD	0.664	<0.001	20.49	0.613	0.616	—	0.406	0.788	0.131
Composite index: MELD-δ (WCC-CRP)	0.707	<0.001	1.89	0.639	0.649	6.48	0.438	0.808	0.124
Composite index: MELD-δ (Bil-WCC)	0.686	<0.001	1.67	0.636	0.638	3.31	0.429	0.804	0.128
Composite index: MELD-centrality	0.701	<0.001	2.30	0.642	0.705	5.57	0.482	0.821	0.123
Composite index: MELD-shortest path	0.709	<0.001	2.23	0.642	0.684	6.78	0.465	0.817	0.122
Composite index: MELD-diameter	0.706	<0.001	2.07	0.632	0.662	6.33	0.445	0.809	0.125

δ, deviation along an axis; Alb, serum albumin; AUC, area under the curve; Bil, total bilirubin; CRP, C-reactive protein; HR, heart rate; INR, international normalized ratio; MELD, model for end-stage liver disease; Na, serum sodium; NPV, negative predictive value; PPV, positive predictive value; WCC, white cell count.

**Table 6.** Measure of prognostic improvement of MELD due to the addition of parenclitic indexes

	IDI	*P* value	NRI	*P* value
MELD + WCC-CRP	0.0359	0.003	0.3384	0.004
MELD + Bil-WCC	0.0157	0.040	0.2146	0.051
MELD + centrality	0.0396	0.002	0.2991	0.012
MELD + shortest path	0.0419	0.001	0.3795	0.001
MELD + diameter	0.0284	0.007	0.3040	0.009

δ, deviation along an axis; Alb, serum albumin; Bil, total bilirubin; CRP, C-reactive protein; IDI, integrated discrimination improvement; INR, international normalized ratio; MELD, model for end-stage liver disease; Na, serum sodium; NRI, net reclassification indexes; WCC, white cell count.

### Assessment of the prognostic value of baseline parenclitic indexes to differentiate between 6-month outcomes comparing standard care with targeted albumin therapy

**Figure 3.**
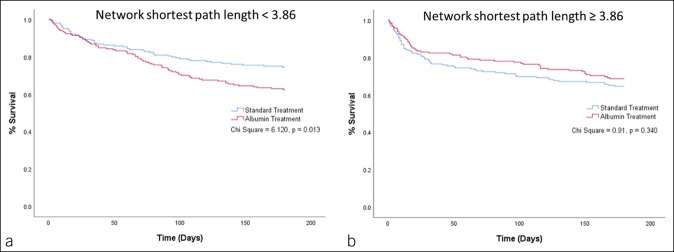
Kaplan-Meier graph representing 6-month survival prediction of patients based on network shortest path length cutoff (**a** vs **b**) and treatment (standard care vs targeted albumin therapy).

**Figure 4.**
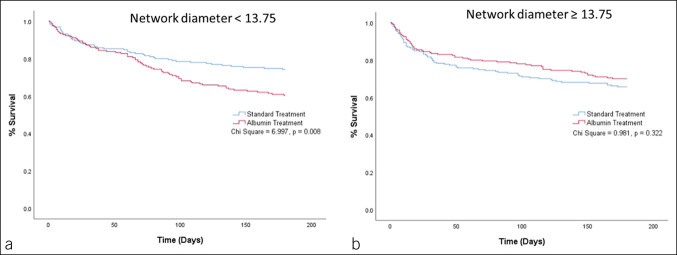
Kaplan-Meier graph representing 6-month survival prediction of patients based on network diameter cutoff (**a** vs **b**) and treatment (standard care vs targeted albumin therapy).

**Figure 5.**
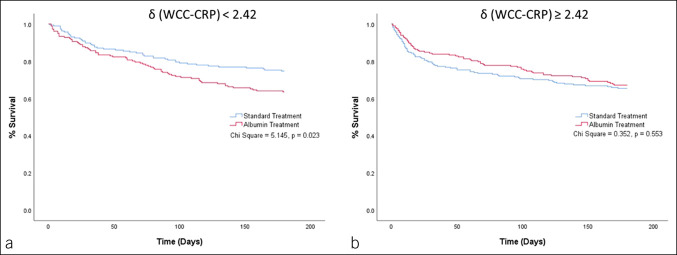
Kaplan-Meier graph representing 6-month survival prediction of patients based on δ (WCC-CRP) cutoff (**a** vs **b**) and treatment (standard care vs targeted albumin therapy). δ, deviation along an axis; CRP, C-reactive protein; WCC, white cell count.

The result of multivariate Cox regression for the interaction of targeted albumin treatment with baseline parenclitic variables to predict 6-month survival showed that measures of global parenclitic network characteristics (i.e., diameter and mean shortest path length), WCC-CRP parenclitic deviation, baseline serum albumin, and white cell count significantly interacted with targeted albumin treatment to predict survival (see Supplementary Table S4, Supplementary Digital Content, http://links.lww.com/CTG/A931). Furthermore, the Kaplan-Meier survival curves for patients grouped by the parenclitic indexes demonstrated significant differences when targeted albumin and standard care groups were compared (Figures 3,4,5). Specifically, patients with lower deviation along the WCC-CRP axis (<2.42) showed consistently lower survival (%) over the whole of the 6-month follow-up period in the targeted albumin arm compared with standard care, not just for the trial treatment period (*P* = 0.023, Figure 5a). The same pattern applied when we considered disruption/deviation in the global parenclitic network, with patients with lower network shortest path length and lower network diameter in standard care showing significantly higher survival rates compared with the targeted albumin group (*P* = 0.013 and 0.008, respectively, Figures 3a,4a). Again, the difference in survival persisted throughout the duration of the 6-month follow-up period. This difference in 6-month survival between treatment groups could not be detected when stratifying by the baseline MELD score, and there was no difference in survival between treatment groups in the overall study population. In patients with higher deviation along the WCC-CRP axis (>2.42), network shortest path length (>3.86), and network diameter (>13.75), there were no differences in survival between treatment groups (Figures 3,4,5b).

## DISCUSSION

Our parenclitic network analyses using baseline clinical data accurately predict outcome in patients with decompensated cirrhosis hospitalized for acute complications, based on disruption of organ system coupling. These analyses also identified that patients with preserved organ system coupling had significantly poorer outcomes after increased albumin treatment.

Reduced organ system correlation was associated with poorer prognosis in hospitalized patients with cirrhosis independent of the severity of disease and age. The dyscoordination in the crosstalk between the key markers of systemic inflammatory response, CRP and WCC, provides a novel pathophysiological insight into the dysregulated inflammatory response in decompensated cirrhosis not captured by the MELD severity scoring system. Specifically, we found that reduced coordination between CRP and WCC predicted poorer 6-month outcomes in patients receiving standard clinical treatment. This was in line with our previous validation study in decompensated cirrhosis (27). Furthermore, the parenclitic network approach used here is based on data that are routinely available anywhere in the world and cost effective while also providing interpretable physiological insights compared with the machine learning or artificial intelligence approach that requires larger sample size or involves an uninterpretable blackbox (31). Compared with the study by Zhang et al (27), our study involves a larger group of patients hospitalized for cirrhotic decompensation who were prospectively recruited and followed up across various hospitals in the United Kingdom. Also, to assess the integrity of the method used, we used a split validation method, which confirmed that the result of the analyses was robust.

Prognostic modeling in cirrhosis from Child-Pugh to MELD-plus and acute-on-chronic liver failure continues to move toward greater recognition of the impact on survival of the extrahepatic involvements of cirrhosis (32–34). A major trigger for this organ system disconnection is inflammation, either pathogen-associated molecular pattern (PAMP) or damage-associated molecular pattern (DAMP) (35,36), with the presence of infection linked to a 4-fold increase in the risk of mortality (37,38). CRP is an acute phase protein that increases in plasma after systemic inflammation and tissue or cell death (39–41). CRP transcription and synthesis is primarily induced by IL-6 and results in the activation of the classical complement pathways and the recruitment of phagocytic cells to the site of infection (42,43). Thus, under physiological condition, the activities of WCC and CRP are closely coordinated toward achieving an effective response to infections. However, because CRP is produced by the hepatocytes in response to inflammation, the use of the serum CRP level as a biomarker of inflammation or infection in cirrhosis has been contested (44–48).

Leukopenia is associated with a significantly higher risk of decompensation and mortality in patients with cirrhosis (48,49). However, there are no established standardized thresholds suitable to accurately distinguish survivors and nonsurvivors in cirrhosis (50). Therefore, both CRP and WCC, although useful markers of infection and inflammation in cirrhosis, have weak prognostic values when considered individually. However, when we considered both biomarkers as a coordinated axis in a network, this significantly predicted survival independent of MELD. The combination of these biomarkers of inflammatory response into a single physiological axis appears to provide a more sophisticated picture of the pathophysiological disturbance in inflammasomes because of cirrhosis.

Unexpectedly, patients with lower parenclitic deviation and by extension higher organ system connectivity showed significantly lower survival after targeted albumin therapy for a maximum of 2 weeks that persisted over the 6-month follow-up. We hypothesize that patients with preserved organ system connectivity may achieve this by the diversion of energies/resources needed for normal physiological functions toward maintaining an effective inflammatory response (51). Targeted albumin therapy for a short period may represent an adverse biological challenge to an already delicately balanced physiological state, which may underlie their significantly poorer prognosis. Conversely, patients with significant disturbances in organ system connectivity appeared to respond slightly better to targeted albumin therapy over the 6-month follow-up period, and this raises the possibility that targeted albumin infusions in these patients improve homeostasis. Perhaps continuation of targeted albumin infusions beyond 2 weeks, which had been shown to have beneficial effects in the ANSWER trial (52), could be further investigated in patients with decompensated cirrhosis, especially those with higher organ system disconnection (see graphical abstract).

The main limitation of this study includes the inherent inability of the parenclitic network to capture the time course of organ system connectivity because of the cross-sectional static feature of the data analyzed. For instance, our analysis is not robust to immediate and temporal changes to the network of organ systems that may follow albumin infusion or result from major clinical events that occur after the baseline data were gathered. Finally, although this study is multicenter in design, it applies to patients with decompensated cirrhosis admitted into hospitals in the United Kingdom where standard care may differ in definition from other countries and regions of the world. Thus, interpretation of our findings should be contextualized. However, the clinical management of patients is still strongly informed by baseline clinical variables, and the ATTIRE study represents one of the largest clinical trials of hospitalized patients with cirrhosis who were prospectively recruited and carefully followed up. The calculation of parenclitic deviation along the WCC-CRP axis is feasible at the bedside. However, further validation of the WCC-CRP axis is required in a larger, globally representative reference group to allow clinical incorporation as a bedside scoring system.

In summary, network analysis using routine clinical data collected at baseline provides novel insights into the pathophysiology of cirrhosis independent of the MELD score and significantly improved MELD's prognostic value. Furthermore, we showed that an unsupervised network analysis has potential value and may predict a poor response to targeted albumin therapy, which was not observed in conventional analyses. Future studies should further investigate the value of WCC-CRP and network mapping to predict outcome in decompensated cirrhosis and improving selection of patients for further trials of albumin or other immune-/inflammation-modulating therapies.

## CONFLICTS OF INTEREST

**Guarantor of the article:** Ali R. Mani, MD, PhD, and Alastair O'Brien, PhD, FRCP.

**Specific author contributions:** T.O., A.O., K.P.M., and A.R.M. contributed to the conceptualization. E.F. and A.O. contributed to clinical data collection. T.O. and A.R.M. contributed to the formal analysis and software development. A.R.M. contributed to the supervision. T.O., A.R.M., A.O., and K.P.M. contributed to the writing—review and editing. All authors contributed to the article and approved the submitted version.

**Financial support:** Wellcome Trust Grant Number (WT102568).

**Potential competing interests:** None to report.

## Supplementary Material
