# Supplementary material for: Parenclitic Network Mapping Identifies Response to Targeted Albumin Therapy in Patients Hospitalized With Decompensated Cirrhosis
Source: Clin Transl Gastroenterol. 2023 Apr 5;14(6):e00587. doi: 10.14309/ctg.0000000000000587 (PMC10299770; doi:10.14309/ctg.0000000000000587)

**Supplementary table S1.**  Description of network topology indices used in this study.

| **Network Topology Measures** | **Definition** |
| --- | --- |
| Mean Centrality | Mean of degree centrality of each node. Degree centrality is an index for the importance of each node and is measured as the sum of weighted incoming or outcoming edges of each node. |
| Mean Shortest path length | Mean of the shortest path length in the network. Shortest path length is a path with the minimum number of edges between two nodes. In a weighted network, it is a path with the minimum sum of edge weights starting at source node and ending at target node. |
| Network diameter | The shortest path length between the most distant nodes. |

**Supplementary table S2.** Differences in parenclitic indices between survivors and non-survivors in the validation group. Survivors in the training group (n=194) were used as a model for calculation of the coefficients that were used for calculation of parenclitic deviations and network indices in the validation group (n=203).

| **Variables** | **Survivor** | **Non-survivors** | **p-value** |
| --- | --- | --- | --- |
| δ (WCC-CRP) | 3.74 (1.17-5.78) | 2.23 (1.17-3.79) | 0.061 |
| **δ (Bil-WCC)** | **4.11 (1.33-7.84)** | **2.66 (1.16-4.28)** | **0.040** |
| **Mean Centrality** | **6.37 (4.09-9.15)** | **5.12 (3.23-7.21)** | **0.013** |
| **Mean Shortest path length** | **6.4 (4.39-10.03)** | **5.18 (3.58-7.53)** | **0.011** |
| **Diameter** | **17.9 (11.52-27.54)** | **13.51 (8.49-20.29)** | **0.017** |

δ*; Deviation along an axis, WCC; White Cell Count, Na; Serum Sodium, Bil; Total Bilirubin,*

**Supplementary table S3.** Prognostic values of parenclitic indices independent of age and MELD in the randomly split (~50%) standard treatment group. During validation, the correlation coefficients were recalculated in a training sample of 194 of randomly selected patients. The individual parenclitic deviations were calculated using these coefficients for the validation sample comprising the remainder 203 patients. Cox regression was used to estimate hazard ratios based on 6-month follow-up survival data.

| **Variables** | **β** | **SEM** | **p-value** | **Hazard Ratio (95%CI)** |
| --- | --- | --- | --- | --- |
| **δ (WCC-CRP)** | **0.098** | **0.049** | **0.048** | **1.102 (1.001-1.214)** |
| Age | 0.047 | 0.014 | 0.001 | 1.048 (1.02-1.077) |
| MELD | 0.088 | 0.019 | <0.001 | 1.092 (1.052-1.133) |
|  |  |  |  |  |
| **δ (Bil-WCC)** | **0.074** | **0.029** | **0.011** | **1.077 (1.017-1.14)** |
| Age | 0.043 | 0.013 | 0.001 | 1.044 (1.017-1.071) |
| MELD | 0.078 | 0.018 | <0.001 | 1.081 (1.044-1.119) |
|  |  |  |  |  |
| Mean Centrality | 0.09 | 0.049 | 0.066 | 1.094 (0.994-1.205) |
| Age | 0.047 | 0.014 | <0.001 | 1.049 (1.021-1.077) |
| MELD | 0.082 | 0.02 | <0.001 | 1.085 (1.043-1.129) |
|  |  |  |  |  |
| **Mean Shortest path length** | **0.086** | **0.042** | **0.042** | **1.09 (1.003-1.184)** |
| Age | 0.047 | 0.014 | 0.001 | 1.048 (1.021-1.077) |
| MELD | 0.083 | 0.02 | <0.001 | 1.086 (1.045-1.129) |
|  |  |  |  |  |
| **Diameter** | **0.03** | **0.014** | **0.034** | **1.03 (1.002-1.059)** |
| Age | 0.047 | 0.013 | <0.001 | 1.049 (1.021-1.077) |
| MELD | 0.084 | 0.019 | <0.001 | 1.088 (1.048-1.13) |

δ*; Deviation along an axis, WCC; White Cell Count, Na; Serum Sodium, Bil; Total Bilirubin,*

**Supplementary table S4.** Interaction between parenclitic indices and albumin administration in predicting 6-month survival.

| **Variables** | **β** | **SEM** | **Hazard Ratio (95%CI)** | **p-value** |
| --- | --- | --- | --- | --- |
| Age | 0.06 | 0.013 | 1.062 (1.037-1.089) | <0.001 |
| TX | -0.451 | 0.408 | 0.637 (0.286-1.418) | 0.269 |
| Age*TX | 0.015 | 0.018 | 1.015 (0.98-1.051) | 0.402 |
|  |  |  |  |  |
| MELD | 0.026 | 0.008 | 1.026 (1.01-1.043) | 0.002 |
| TX | -0.738 | 0.68 | 0.478 (0.126-1.812) | 0.278 |
| MELD*TX | 0.011 | 0.012 | 1.011 (0.988-1.035) | 0.357 |
|  |  |  |  |  |
| Gender | -0.203 | 0.194 | 0.816 (0.558-1.194) | 0.296 |
| TX | -0.38 | 0.383 | 0.684 (0.323-1.449) | 0.321 |
| Gender*TX | 0.187 | 0.285 | 1.205 (0.69-2.106) | 0.512 |
|  |  |  |  |  |
| Diameter | 0.005 | 0.01 | 1.005 (0.985-1.025) | 0.64 |
| TX | -0.573 | 0.246 | 0.564 (0.348-0.913) | 0.02 |
| **Diameter*TX** | **0.026** | **0.013** | **1.026 (1.00-1.052)** | **0.049** |
|  |  |  |  |  |
| Mean Shortest path | 0.058 | 0.042 | 1.06 (0.976-1.151) | 0.167 |
| TX | -0.657 | 0.273 | 0.518 (0.303-0.886) | 0.016 |
| **Mean Shortest path *TX** | **0.118** | **0.057** | **1.125 (1.005-1.259)** | **0.040** |
|  |  |  |  |  |
| Mean Centrality | 0.057 | 0.026 | 1.059 (1.007-1.114) | 0.027 |
| TX | -0.552 | 0.288 | 0.576 (0.327-1.013) | 0.055 |
| Mean Centrality *TX | 0.053 | 0.036 | 1.054 (0.983-1.131) | 0.14 |
|  |  |  |  |  |
| δ (Bil-WCC) | 0.017 | 0.022 | 1.017 (0.973-1.063) | 0.459 |
| TX | -0.369 | 0.175 | 0.691 (0.49-0.975) | 0.035 |
| δ (Bil-WCC)*TX | 0.06 | 0.031 | 1.062 (0.999-1.128) | 0.053 |
|  |  |  |  |  |
| δ (WCC-CRP) | -0.001 | 0.021 | 0.999 (0.958-1.042) | 0.973 |
| TX | -0.572 | 0.181 | 0.565 (0.396-0.804) | 0.002 |
| **δ (WCC-CRP)*TX** | **0.122** | **0.033** | **1.13 (1.058-1.206)** | **<0.001** |
|  |  |  |  |  |
| Alb | -0.078 | 0.023 | 0.925 (0.884-0.967) | 0.001 |
| TX | -1.909 | 0.776 | 0.148 (0.032-0.678) | 0.014 |
| **Alb*TX** | **0.078** | **0.034** | **1.081 (1.012-1.154)** | **0.021** |
|  |  |  |  |  |
| WCC | 0.026 | 0.015 | 1.027 (0.997-1.057) | 0.081 |
| TX | -0.671 | 0.247 | 0.511 (0.315-0.829) | 0.006 |
| **TX*WCC** | **0.056** | **0.021** | **1.058 (1.015-1.103)** | **0.007** |
|  |  |  |  |  |
| CRP | 0.002 | 0.001 | 1.002 (1-1.004) | 0.069 |
| TX | -0.37 | 0.161 | 0.691 (0.504-0.948) | 0.022 |
| **CRP*TX** | **0.006** | **0.002** | **1.006 (1.002-1.01)** | **0.003** |

δ*; Deviation along an axis, WCC; White Cell Count, Na; Serum Sodium, Bil; Total Bilirubin, INR; International Normalized Ratio, Alb; Serum Albumin, HR; Heart Rate, CRP; C-Reactive Protein, Cent; Centrality, SPL; Shortest Path Length, MELD; Model for End-stage Liver Disease, TX; Treatment.*

**Supplementary figure S1.** Network map of the 6-month survivors in the randomly split standard treatment group (training sample).


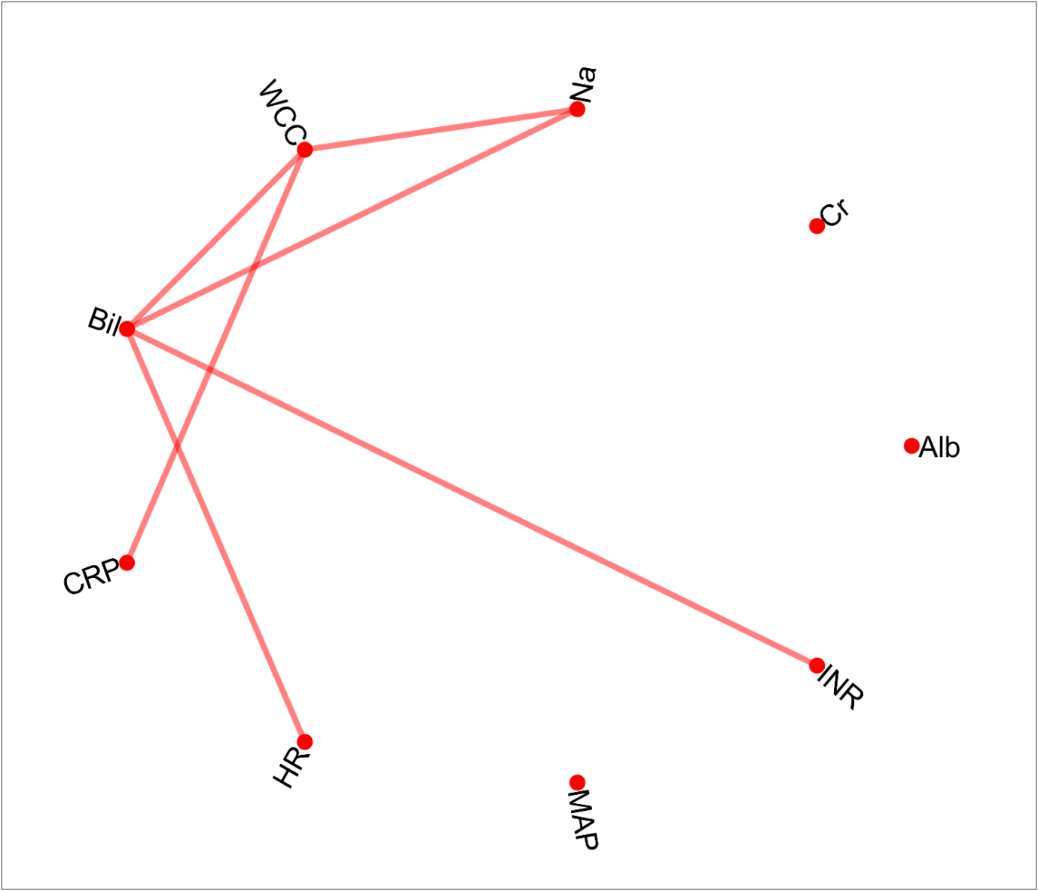


**Supplementary figure S2.** Network map of the 6-month non-survivors in the randomly split standard treatment group (training sample).


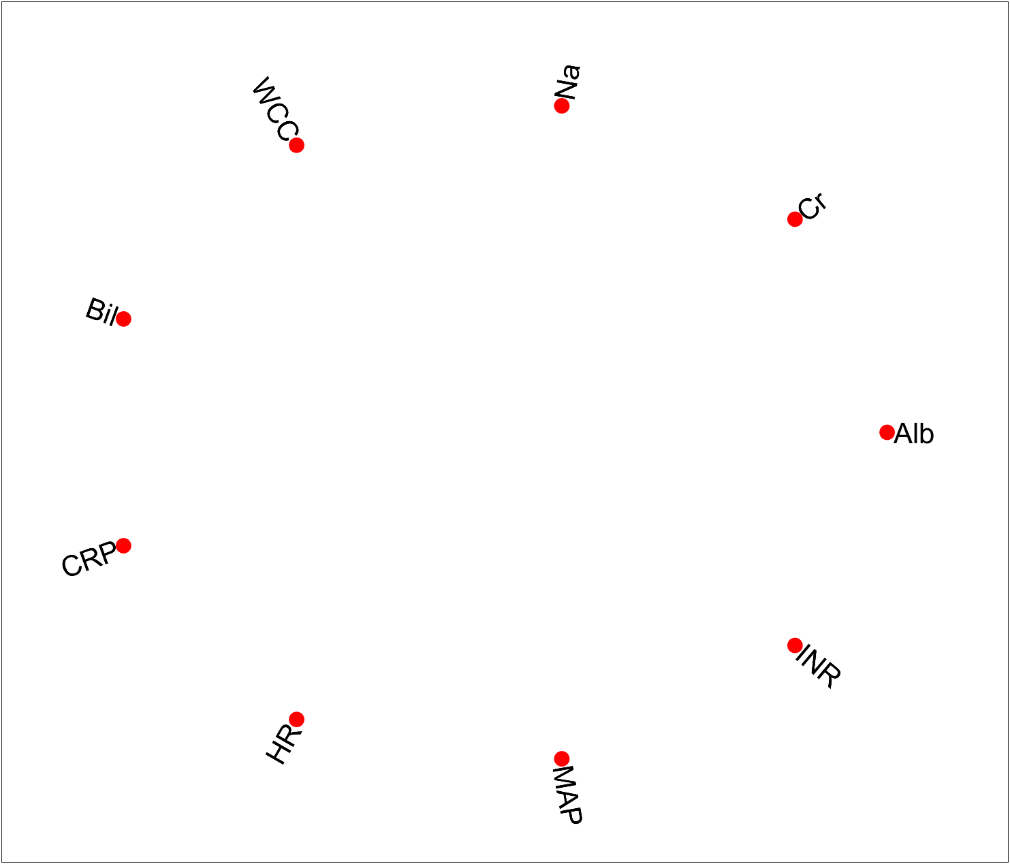

Supplement: Supplementary file 1 [file ct9-14-e00587-s001.docx]
